# Growth of NiAl‐Layered Double Hydroxide on Graphene toward Excellent Anticorrosive Microwave Absorption Application

**DOI:** 10.1002/advs.202002658

**Published:** 2021-01-06

**Authors:** Xuefei Xu, Shaohua Shi, Yulin Tang, Guizhen Wang, Maofan Zhou, Guoqing Zhao, Xuechun Zhou, Shiwei Lin, Fanbin Meng

**Affiliations:** ^1^ State Key Laboratory of Advanced Materials of Tropical Island Resources (Ministry of Education) Hainan University Haikou Hainan 570228 P. R. China; ^2^ State Key Laboratory of Advanced Technologies of Materials (Ministry of Education) School of Materials Science and Engineering Southwest Jiaotong University Chengdu Sichuan 610031 P. R. China

**Keywords:** anticorrosion, atomic layer deposition (ALD), double layered hydroxides (LDHs), graphene, microwave absorption

## Abstract

High‐performance microwave absorbers with special features are desired to meet the requirements of more complex modern service environments, especially corrosive environments. Therefore, high‐efficiency microwave absorbers with corrosion resistance should be developed urgently. Herein, a 3D NiAl‐layered double hydroxide/graphene (NiAl‐LDH/G) composite synthesized by atomic‐layer‐deposition‐assisted in situ growth is presented as an anticorrosive microwave absorber. The content of NiAl‐LDH in the composite is optimized to achieve impedance matching. Furthermore, under the cooperative effects of the interface polarization loss, conduction loss, and 3D porous sandwich‐like structure, the optimal NiAl‐LDH/G shows excellent microwave absorption performance with a minimum reflection loss of −41.5 dB and a maximum effective absorption bandwidth of 4.4 GHz at a loading of only 7 wt% in epoxy. Remarkably, the encapsulation effect of NiAl‐LDH can restrain the galvanic corrosion owing to graphene. The epoxy coating with the NiAl‐LDH/G microwave absorber on carbon steel exhibits long‐term corrosion resistance, owing to the synergetic effect of the superior impermeability of graphene and the chloridion‐capture capacity of the NiAl‐LDH. The NiAl‐LDH/G composite is a promising anticorrosive microwave absorber, and the findings of this study may motivate the development of functional microwave absorbers that meet the demands of anticorrosive performance of coatings.

## Graphical abstract

NiAl‐layered double hydroxide/graphene (NiAl‐LDH/graphene) synthesized by atomic layer deposition‐assisted in situ growth is presented as an anticorrosive microwave absorber. The NiAl‐LDH/graphene shows excellent microwave absorption performance with a minimum reflection loss of −41.5 dB and an effective absorption bandwidth of 4.4 GHz. Furthermore, the NiAl‐LDH/graphene coating can exhibit a long‐term corrosion protection for carbon steel.

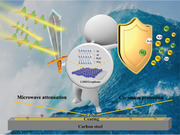

## 1 Introduction

With the rapid development of electromagnetic techniques, serious electromagnetic pollution has arisen, imposing an urgent demand for microwave absorption (MA) materials.^[^ ^1^, ^2^, ^3^, ^4^ ^]^ In the past decades, researchers have made significant efforts to improve the reflection loss (RL) and effective absorption bandwidth (EAB) of microwave absorbers by regulating their composition and microstructure.^[^ ^5^, ^6^ ^]^ However, in practical applications, the complexity and harshness of the environment impose more challenges on the absorbers, and their environmental suitability is therefore of importance. Typically, in the ocean environment, the exfoliation of coatings owing to metal corrosion can invalidate the MA performance of the weapon such as naval vessels and submarine.^[^ ^7^, ^8^ ^]^ Thus, endowing MA materials with corrosion resistance is very important and necessary.

Graphene has received considerable interest in the development of MA materials owing to its high conductive loss and dielectric loss, which can effectively transform the electromagnetic energy into thermal energy.^[^ ^9^, ^10^, ^11^ ^]^ Moreover, specific 2D lamellar structures can introduce cross‐linked electric loss networks, and induce multiple internal reflections and scattering as well as capacitor‐like interactions. They can also accelerate the attenuation of incident microwaves.^[^ ^12^ ^]^ In addition, owing to its impermeability and chemical stability, graphene has been proposed as a perfect atomic‐scale barrier to prevent metal substrates from corrosion. Prasai et al. reported that the corrosion rate of graphene‐coated Ni is 20 times slower than that of bare Ni, indicating the excellent anticorrosive effect of graphene.^[^ ^13^ ^]^ Dong et al. deposited multilayered graphene on the surface of Cu and studied its electrochemical performance in an aqueous NaCl solution. The results revealed that the graphene coating could provide 14 days of corrosion protection to Cu.^[^ ^14^ ^]^ Chang et al. demonstrated that embedding well‐dispersed graphene nanosheets in an epoxy coating could increase the corrosion potential to 0.4 V.^[^ ^15^ ^]^ In summary, graphene is one of the most promising candidates for anticorrosive MA materials. However, the high conductivity of pure graphene impedes its extensive application in the field of microwave absorption and corrosion resistance. According to Maxwell's theory, excessive conductive loss might lead to impedance mismatch, causing an increase in the microwave reflection rather than absorption.^[^ ^16^, ^17^ ^]^ Wang et al. showed that the RL of pure graphene is no less than −5 dB over the entire testing frequency (2–18 GHz), which is far from the requirement of practical applications.^[^ ^18^ ^]^ In addition, researchers have found that pure graphene could only provide short‐term protection for the metal substrate. The rate of corrosion could be accelerated with time because the galvanic couple formed between the conductive graphene and metal substrate would lead to galvanic corrosion at the damaged areas of graphene.^[^ ^19^, ^20^ ^]^

In this work, NiAl‐layered double hydroxide (NiAl‐LDH) nanoflakes grown on a graphene surface were designed and prepared with the assistance of an atomic layer deposition (ALD) approach. The complex 3D structure with the low‐conductivity NiAl‐LDH can remedy the disadvantages of the highly conductive pure graphene. First, good impedance matching and high‐performance microwave absorption can be achieved by regulating the content of NiAl‐LDH in the composite. Second, the encapsulation effect of NiAl‐LDH can passivate the graphene surface, thus reducing the risk of galvanic corrosion. Third, because of its outstanding chloride (Cl^−^)‐capturing capacity, NiAl‐LDH can decrease the permeation of Cl^−^, thereby improving the corrosion resistance of the coating. Therefore, this highly complex 3D structure can not only increase the opportunity for microwave scattering, but also prolong the infiltration path of the corrosive medium. Owing to the precise component regulation and ingenious structural design, the optimal NiAl‐LDH/graphene (100 NiAl‐LDH/G) exhibits excellent microwave absorption performance with a minimum RL of −41.5 dB, and a maximum EAB of 4.4 GHz is obtained at a loading of only 7 wt%. In addition, the electrochemical results suggest that the 7 wt% NiAl‐LDH/G‐epoxy coating on carbon steel can have long‐term corrosion protection performance. Thus, the as‐obtained NiAl‐LDH/G can be regarded as a prospective anticorrosive microwave absorber. The findings of this study can also pave the way for the design of high‐performance microwave absorbers with strong corrosion protection performance.

## 2 Results and Discussion

### 2.1 Characterization

**Figure 1:**
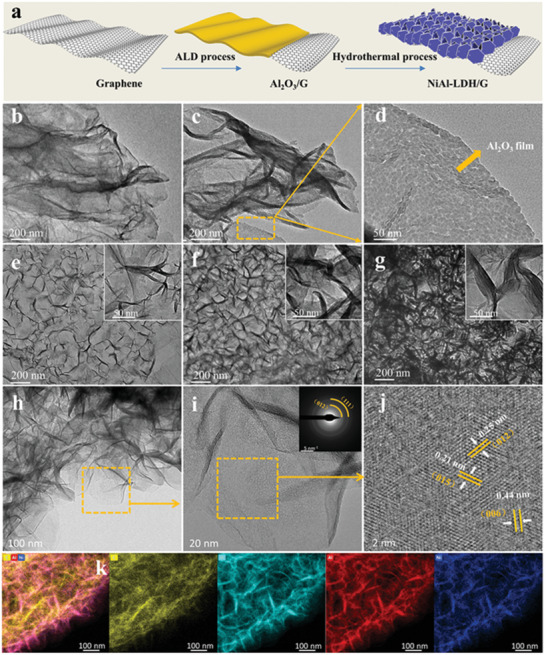
a) Schematic illustration of the preparation process of NiAl‐LDH/G. TEM images of b) graphene, c,d) Al_2_O_3_/G, e) 50 NiAl‐LDH/G, f) 100 NiAl‐LDH/G, and g) 150 NiAl‐LDH/G, respectively. h,i) TEM and j) HRTEM images of NiAl‐LDH nanoflakes in the 100 NiAl‐LDH/G sample. k) Elemental maps of C, O, Al, and Ni in the 100 NiAl‐LDH/G sample.

The fabrication process of hierarchical NiAl‐LDH/G is shown in **Figure** **1**a. In the first step, a uniform Al_2_O_3_ film is deposited on the graphene surface by the ALD process, and the film thickness can be controlled easily by adjusting the number of ALD cycles. Then, through an in situ growth procedure, the NiAl‐LDH nanoflakes are generated by the reaction of Al^3+^ decomposed from Al_2_O_3_ with Ni^2+^ under alkaline conditions. The decomposition of Al_2_O_3_ and the growth of the NiAl‐LDH occurred simultaneously, guaranteeing the uniform distribution of the NiAl‐LDH nanoflakes on the graphene surface.^[^ ^21^ ^]^ In order to investigate the effect of the Al_2_O_3_ content on the morphology of NiAl‐LDH, NiAl‐LDH/G samples deposited with 50, 100, and 150 ALD cycles of Al_2_O_3_ were prepared and denoted as 50‐, 100‐, and 150 NiAl‐LDH/G, respectively.

The microstructures of graphene and Al_2_O_3_/G were characterized by transmission electron microscopy (TEM) (Figure 1b–d). Initially, graphene presents a characteristic 2D layer structure with many wrinkles. After the deposition of Al_2_O_3_ through 100 ALD cycles, a thin Al_2_O_3_ film formed on both sides of graphene. The Al_2_O_3_ film was very uniform and continuous even at the wrinkles of graphene, indicating the step coverage of the ALD method. The scanning electron microscopy (SEM) images (Figure S1, Supporting Information) show that the ultrathin NiAl‐LDH nanoflakes are distributed vertically on both sides of graphene, and they intersect each other uniformly, forming an interesting 3D porous array structure. The formation of the 3D structure can be explained as follows: during the in situ growth of NiAl‐LDH, Al^3+^ obtained by the decomposition of Al_2_O_3_ first adsorbed onto the graphene surface and reacted with Ni^2+^ under the alkaline conditions to generate NiAl‐LDH with a positive charge. Owing to the strong electrostatic attraction, the initial NiAl‐LDH grew parallel to the negatively charged graphene substrate. Subsequently, the NiAl‐LDH tended to grow vertically on the initially formed NiAl‐LDH/G to decrease the electrostatic repulsion between the positively charged NiAl‐LDH laminates and NiAl‐LDH/G.^[^ ^22^ ^]^ This 3D structure can result in a large surface area and extra void space, which is conducive for microwave scattering. Moreover, the amount of NiAl‐LDH nanoflakes increased with increasing Al_2_O_3_ content, as observed more clearly in the TEM images (Figure 1e–g). In the case of 50 NiAl‐LDH/G, only a few NiAl‐LDH nanoflakes adhered to the graphene surface are observed. This might be because the Al^3+^ concentration in the reaction solution is insufficient. With the increase in the cycle number of Al_2_O_3_ ALD, both the size and the content of the NiAl‐LDH nanoflakes increased. However, when the cycle number of Al_2_O_3_ ALD reached 150, the NiAl‐LDH nanoflakes began to aggregate, resulting in reduced void spaces. Thus, the morphology of NiAl‐LDH/G can be regulated readily by controlling the cycle number of Al_2_O_3_ ALD. The crystal structure of NiAl‐LDH obtained with 100 ALD cycles of Al_2_O_3_ was further studied by high‐resolution (HR) TEM and selected area electron diffraction (SAED). As shown in Figure 1h, the average size of the ultrathin NiAl‐LDH nanoflake is ≈100 nm, and the nanoflakes are distributed on the graphene surface compactly with random orientation. The SAED pattern (inset in Figure 1i) presents several rings, demonstrating the crystalline structure of NiAl‐LDH. Furthermore, the highlighted lattice fringes in the HRTEM images (Figure 1j) with spacings of 0.21, 0.25, and 0.44 nm can be assigned to the (015), (012), and (006) planes of NiAl‐LDH, respectively. In addition, the energy‐dispersive X‐ray spectroscopic (EDS) maps (Figure 1k) indicate a uniform distribution of C, O, Al, and Ni in NiAl‐LDH/G, further suggesting that the NiAl‐LDH nanoflakes are distributed uniformly on the graphene surface.

**Figure 2:**
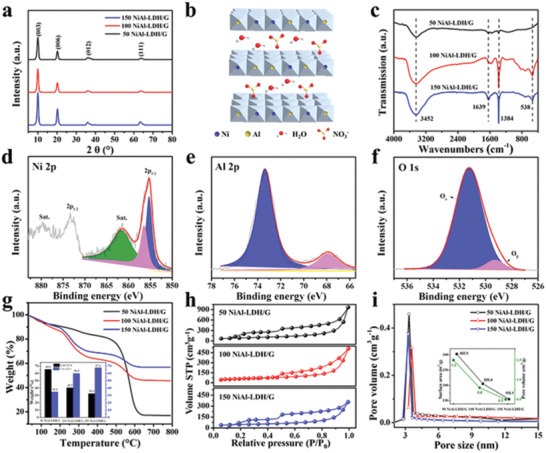
a) XRD patterns of NiAl‐LDH/G samples. b) Crystal structure of NiAl‐LDH‐NO_3_^−^. c) FTIR analysis of NiAl‐LDH/G samples. High‐resolution XPS profiles of d) Ni 2p, e) Al 2p, and f) O1s for the 100 NiAl‐LDH/G sample. g) TG curves in the range of 30–800 °C under air and the weight ratio between NiAl‐LDH and graphene in the NiAl‐LDH/G samples. h) BET surface area and i) pore size distribution of NiAl‐LDH/G samples.

The crystal structures of the Al_2_O_3_/G and NiAl‐LDH/G samples were further analyzed by X‐ray diffraction (XRD). For Al_2_O_3_/G (Figure S2a, Supporting Information), only a single broad diffraction peak is observed at *2θ* = 24.2°, corresponding to graphene. This can be ascribed to the amorphous Al_2_O_3_ film prepared at the low temperature (150 °C), which yields no XRD peaks. For the NiAl‐LDH/G samples (**Figure** **2**a), a series of diffraction peaks at *2θ* = 10.0°, 20.0°, 35.9°, and 63.1° that can be indexed to the (003), (009), (012), and (111) planes of NiAl‐LDH‐NO_3_^−^ are observed, agreeing with the TEM results.^[^ ^23^ ^]^ The results demonstrate that layered NiAl‐LDH intercalated with NO_3_^−^ (see the structural diagram in Figure 2b) was successfully grown on graphene. In addition, the absence of the graphene peak in the NiAl‐LDH/G samples may be due to the lower crystallinity of graphene compared to that of NiAl‐LDH.

Figure 2c shows the Fourier‐transform infrared (FTIR) spectra of 50 NiAl‐LDH/G, 100 NiAl‐LDH/G, and 150 NiAl‐LDH/G. The peaks at 3452 and 1639 cm^−1^ can be ascribed to the O—H stretching and bending vibrations of the adsorbed water molecules. The peak at 1384 cm^−1^ is due to the stretching vibration of NO_3_^−^ intercalated in the LDH layers, and the characteristic peak at 538 cm^−1^ is due to the stretching and bending modes of the metal oxides (Ni—O and Al—O), confirming the formation of NiAl‐LDH‐NO_3_^−^.^[^ ^24^ ^]^

X‐ray photoelectron spectroscopy (XPS) was performed to determine the detailed chemical states of the as‐prepared NiAl‐LDH/G. The XPS survey profiles (Figure S2b, Supporting Information) display the peaks of C, O, Ni, and Al for all the NiAl‐LDH/G samples, revealing the presence of graphene and NiAl‐LDH in the composites. In the Ni 2p spectrum (Figure 2d), the two peaks at 855.4 and 873.3 eV can be assigned to Ni 2p_2/3_ and Ni 2p_1/2_, respectively. Further, the additional satellite peaks indicate the presence of a high‐spin divalent state of Ni, in agreement with the Ni^2+^ oxidation state. In addition, the high‐resolution Ni 2p_2/3_ spectrum can be fitted with two peaks. The peaks at 855.2 and 856.5 eV are attributed to Ni^2+^ with saturated coordination and unsaturated coordination, respectively.^[^ ^25^ ^]^ The high‐resolution Al 2p spectrum (Figure 2e) confirms the Al^3+^ oxidation state in NiAl‐LDH. The two fitted peaks at 529.3 and 531.2 eV in the high‐resolution O 1s spectrum (Figure 2f) are attributed to hydroxyl groups and NO_3_^−^, respectively.^[^ ^25^ ^]^ All these results indicate the successful fabrication of the hierarchical NiAl‐LDH/G structure.

In order to explore the precise weight ratio of NiAl‐LDH in the composites, thermogravimetric analysis (TGA) was performed from 30 to 800 °C under an air atmosphere. As shown in Figure 2g, the weight loss before 200 °C can be attributed to the decomposition of physically adsorbed water and interlayer water molecules of NiAl‐LDH. The dramatic weight loss in the range of 200–500 °C is ascribed to the pyrolysis of graphene. The weight loss after 500 °C is due to the conversion of NiAl‐LDH to metal oxides. Based on the pyrolytic process, the precise quantity of NiAl‐LDH in the composites was calculated, and is presented in the inset of Figure 2g. The NiAl‐LDH contents in 50 NiAl‐LDH/G, 100 NiAl‐LDH/G, and 150 NiAl‐LDH/G are determined to be 34.4%, 59.5%, and 67.4%, respectively, presenting an obvious increasing trend with increasing cycle number of Al_2_O_3_ ALD.

The Brunner–Emmet–Teller (BET) surface area and porosity of the as‐prepared NiAl‐LDH/G were examined by the analyses of their N_2_ absorption–desorption isotherms (Figure 2g,h). For all three samples, typical type IV isotherms were obtained, suggesting the presence of a porous structure. Furthermore, the pore size distribution of all samples was mainly at ≈4 nm, indicating that the NiAl‐LDH/G samples are mesoporous. The BET surface areas of the 50 NiAl‐LDH/G, 100 NiAl‐LDH/G, and 150 NiAl‐LDH/G are 302.5, 205.4, and 155.1 m^2^ g^−1^, and their pore volumes are 1.5, 0.8, and 0.5, respectively. The reduction in the BET surface and pore volume with increasing ALD cycles should be due to the aggregation of NiAl‐LDH nanoflakes.

### 2.2 The Microwave Absorption Performance

**Figure 3:**
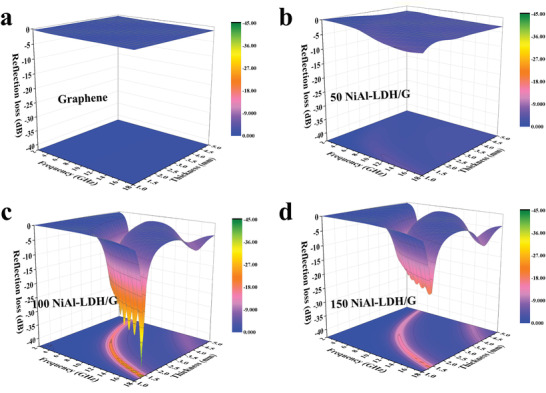
a–d) 3D representations of the RL of a) Graphene, b) 50 NiAl‐LDH/G, c) 100 NiAl‐LDH/G, and d) 150 NiAl‐LDH/G.

**Figure 4:**
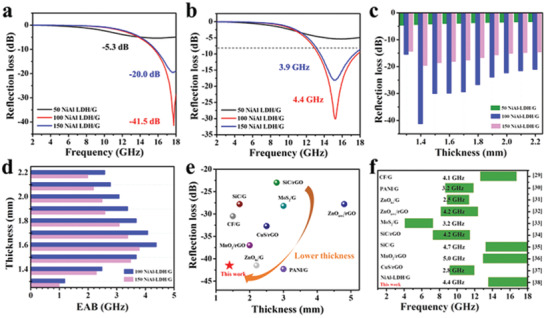
The RL values of 50 NiAl‐LDH/G, 100 NiAl‐LDH/G, and 150 NiAl‐LDH/G at the thickness of a) 1.4 mm and b) 1.6 mm. c) Minimum RL and d) maximum EAB values of NiAl‐LDH/G samples with thicknesses of 1.4–2.2 mm. Comparison of e) the minimum microwave RL and f) the maximum EAB with those of other graphene‐based absorbers reported previously.

To investigate the effect of the NiAl‐LDH content on the microwave absorption properties of the composite, the RL values of graphene, 50 NiAl‐LDH/G, 100 NiAl‐LDH/G, and 150 NiAl‐LDH/G were investigated according to the transmission line theory.^[^ ^26^, ^27^ ^]^ The calculated 3D RL values of the absorbers with different thicknesses of 1–5 mm are shown in **Figure** **3**. Pure graphene displays poor microwave absorption with RL values no less than −5 dB. This can be attributed to the impedance mismatch originating from the high dielectric loss and low magnetic loss, which results in higher microwave reflection from the graphene surface rather than absorption.^[^ ^28^ ^]^ The electromagnetic parameters of pure graphene are presented in Figure S3a–c (Supporting Information). However, after compositing with NiAl‐LDH, an obvious improvement in the microwave absorption performance was observed. For 100 NiAl‐LDH/G and 150 NiAl‐LDH/G, the RL values were below −10 dB over the entire test frequency range upon adjusting the filler thickness. Furthermore, the microwave absorption performance can also be tailored by tuning the content of NiAl‐LDH. As shown in **Figure** **4**a,b, the sample with optimal NiAl‐LDH content, 100 NiAl‐LDH/G, exhibits an excellent microwave absorption performance with a minimum RL value of −41.5 dB at the frequency of 17.8 GHz, and a maximum EAB (RL ←10 dB) of 4.4 GHz, in the frequency range of 13.4–17.8 GHz. It is well known that apart from a strong RL and broad EAB, the thinness of the coating is also a key factor for ideal microwave absorbers. Figure 4c,d compares, in detail, the microwave absorption performances of as‐prepared NiAl‐LDH/G with thicknesses of 1.2–2.2 mm. It is found that 100 NiAl‐LDH/G and 150 NiAl‐LDH/G show excellent microwave absorption properties at a small thickness. Specifically, their RL values are less than −20 dB at 1.4–2.2 mm thickness, and the EABs are more than 3 GHz at 1.5–1.8 mm thickness, indicating that they can be promising lightweight absorber candidates. In particular, 100 NiAl‐LDH/G exhibits the minimum RL value at a thickness of 1.4 mm and the broadest EAB at a thickness of 1.6 mm, which are obviously smaller than the thicknesses reported for most of the graphene‐based absorbers (Figure 4e,f).^[^ ^29^, ^30^, ^31^, ^32^, ^33^, ^34^, ^35^, ^36^, ^37^ ^]^

**Figure 5:**
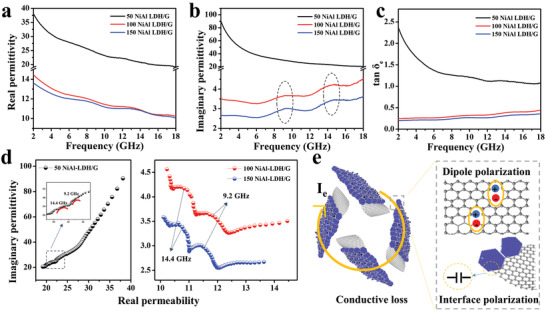
Frequency dependence of the a) real permittivity (*ε*′), b) imaginary permittivity (*ε*″), and c) dielectric loss tangent (tan *δ*_e_). d) Cole–Cole plots of NiAl‐LDH/G samples. e) Schematic of the dielectric loss mechanism in NiAl‐LDH/G absorbers.

In order to understand the reasons for the diverse microwave absorption performances of the different NiAl‐LDH/G samples, the electromagnetic parameters were analyzed (**Figure** **5**a,b). For dielectric loss absorbers, the microwave attenuation ability depends mainly on their complex permittivity.^[^ ^38^, ^39^ ^]^ The real permittivity (*ε*′) represents the ability of the absorber to store the electrical energy, while the imaginary permittivity (*ε*″) refers to its electrical‐energy dissipation capability.^[^ ^40^, ^41^ ^]^ As shown in Figure 5a, with increasing frequency, the *ε*′ values of 50 NiAl‐LDH/G, 100 NiAl‐LDH/G, and 150 NiAl‐LDH/G decreased from 38.1 to 19.1, from 14.5 to 10.2, and from 13.6 to 10.1, respectively. Meanwhile, the *ε*″ values (Figure 5b) of 50 NiAl‐LDH/G decreased gradually from 90.4 to 20.5, and those of 100 NiAl‐LDH/G and 150 NiAl‐LDH/G varied in the range of 3.6–4.5 and 2.5–3.5, respectively. According to the free electron theory (*ε*″ *≈ σ*/2*πε*_0_ *f*), the *ε*″ values correlate positively with the electrical conductivity (*σ*). Thus, with the increase in the NiAl‐LDH content, the *ε*″ values of the NiAl‐LDH/G samples decrease. This is because the content of conductive graphene in the composites decreases with increasing NiAl‐LDH content. Notably, two wide resonance peaks can be observed in the *ε*″ curves, which may be derived from dipole polarization and interface polarization. This can be further confirmed by the Cole–Cole plots. Cole–Cole plots express the relationship between *ε*′ and *ε*″, and each semicircle corresponds to one Debye relaxation process.^[^ ^42^ ^]^ As shown in Figure 5d, all three samples show two obvious semicircles at 9.2 and 14.4 GHz, consistent with the frequencies of the resonance peaks in the *ε*″ curves. For the NiAl‐LDH/G composites, the dipole polarization is due to the asymmetric distribution of charges at the defects or functional groups on graphene, and the electron accumulation at the boundaries of graphene/NiAl‐LDH may result in interface polarization.^[^ ^43^ ^]^ Apart from the two semicircles, the Cole–Cole plots also present an obvious straight line in the tail, indicating that the conductive loss originating from the conductive graphene network in the NiAl‐LDH/G absorbers should not be neglected.^[^ ^44^, ^45^ ^]^ These results indicate that the dielectric loss of NiAl‐LDH/G originates mainly from conductive loss and relaxation loss (dipole polarization and interface polarization) (Figure 5e).

The dielectric loss tangent (tan *δ*_e_ = *ε*″/*ε*′) is also an effective parameter for assessing the dielectric loss capability of absorbers. According to previous reports, a moderate tan *δ*_e_ value is vital for microwave absorption. When the tan *δ*_e_ value of an absorber is more than 1, poor impedance mismatching would deteriorate the absorption.^[^ ^46^ ^]^ As shown in Figure 5c, the tan *δ*_e_ values of 50 NiAl‐LDH/G decrease from 2.4 to 1.1 in the frequency range of 2–18 GHz. Thus, excessive dielectric loss may be responsible for its poor microwave absorption performance. In addition, the real and imaginary permeabilities (*µ*″ and *µ*′) of the NiAl‐LDH/G samples are ≈1 and ≈0, respectively, and the magnetic loss tangent (tan *δ*_m_) is ≈0 (Figure S3d–f, Supporting Information). These values indicate that the microwave absorption performance of NiAl‐LDH/G samples can hardly rely on magnetic loss.

**Figure 6:**
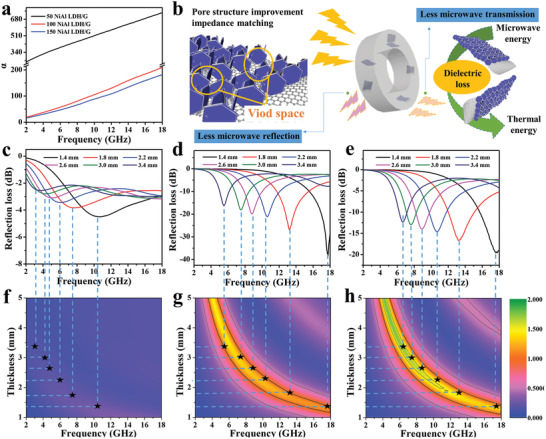
a) Attenuation constants of NiAl‐LDH/G samples. b) Schematic of the microwave attenuation mechanism of NiAl‐LDH/G. The reflection loss and impedance matching characteristics (*Z* = *Z*_in_/*Z*_0_) of c,f) 50 NiAl‐LDH/G, d,g) 100 NiAl‐LDH/G, and e,h) 150 NiAl‐LDH/G at different layer thicknesses.

An ideal microwave absorber should possess strong microwave attenuation ability and good impedance matching synchronously. The inherent microwave attenuation ability can be expressed by the attenuation constant (*α*).^[^ ^47^ ^]^ As shown in **Figure** **6**a, the *α* values of the absorbers increase with increasing frequency, and decrease with increasing NiAl‐LDH content. Figure 6c–h shows the RL and the calculated *Z* values of the NiAl‐LDH/G samples at different thicknesses. According to previous reports, when the *Z* value is close to 1, the microwave can enter the absorber easily and then be converted to thermal energy or other forms of energy.^[^ ^48^, ^49^ ^]^ It can be seen that the *Z* values corresponding to the minimum RL of 100 NiAl‐LDH/G are very close to 1, while those of 50 NiAl‐LDH/G and 150 NiAl‐LDH/G are ≈0.4 and ≈1.5, respectively, indicating that 100 NiAl‐LDH/G achieved the best impedance matching. This good impedance matching of 100 NiAl‐LDH/G can be ascribed to its moderate dielectric loss and high specific surface area. All these results demonstrate that the moderate attenuation ability and good impedance matching are two prerequisites for NiAl‐LDH/G to function as an ideal microwave absorber (Figure 6b).

Additionally, the void spaces introduced by NiAl‐LDH nanoflakes are also beneficial for improving the microwave absorption performance. According to the Maxwell–Garnett theory, a porous material consisting of solid and void components can be considered as an effective electromagnetic medium. The effective permittivity ($\varepsilon_{\mathrm{eff}}^{\mathrm{MG}}$) of such a medium is positively related to its pore volume.^[^ ^50^ ^]^ Therefore, the abundant void spaces of NiAl‐LDH/G can effectively reduce the complex permittivity, thus facilitating impedance matching. Furthermore, abundant active surfaces can be formed within the gaps between the NiAl‐LDH flakes, which lengthen the traveling distances of microwaves due to the multiple reflected propagation paths inside the hybrid materials.^[^ ^51^, ^52^, ^53^ ^]^ Therefore, such unique 3D structures can induce multiple scattering events, thereby increasing the probability of microwave absorption. As discussed above, the excellent microwave absorption performance of 100 NiAl‐LDH/G could be ascribed to its moderate attenuation ability, good impedance matching, and structural effects.

### 2.3 Anticorrosion Property

For application in an ocean environment, microwave absorbers should not only have excellent microwave absorption performance, but also possess strong anticorrosion capability. In general, the anticorrosion capabilities of coatings are evaluated by an electrochemical measurement technique using a three‐electrode system, and the working electrodes are immersed in a 3.5 wt% NaCl solution for simulating the salinity of seawater. The variation in the open‐circuit potential (OCP) values of different steel samples with immersion times was studied to investigate their corrosion behaviors.^[^ ^54^ ^]^ According to Figure S4a (Supporting Information), bare steel has the lowest OCP of ≈−0.70 V. After the deposition of a neat epoxy coating, the OCP of the coated steel shifted in the positive direction, indicating that the epoxy coating can effectively inhibit the corrosion of steel. Interestingly, the OCP declined from −0.56 to −0.60 V in the initial 3 days, and then increased slightly with a further increase in the immersion time. The former is likely due to the permeation of the electrolyte, while the latter is ascribed to the fact that the corrosion products on the steel surface may prevent further contact between the electrolyte and steel. For the carbon steel with an epoxy coating incorporated with 7 wt% NiAl‐LDH/G, the OCP shifted further to a value of −0.27 V, suggesting the lowest tendency of corrosion. This is because NiAl‐LDH/G can fill the tiny pores of the epoxy coating, thus reducing the permeation of the electrolyte.

**Figure 7:**
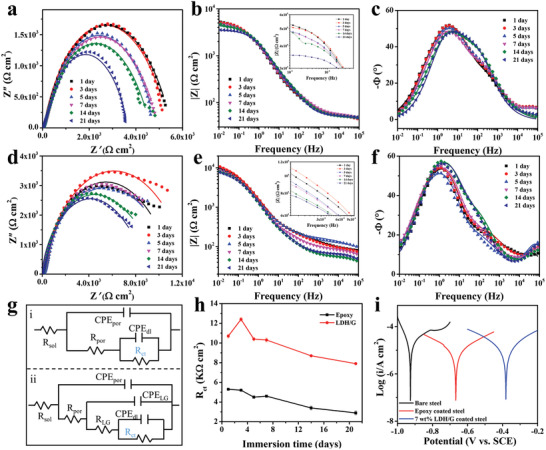
a) Nyquist plots, b) impedance–frequency Bode plots, and c) phase–frequency Bode plots of pure epoxy‐coated steel. d) Nyquist plots, e) impedance–frequency Bode plots, and f) phase–frequency Bode plots of 7 wt% NiAl‐LDH/G/epoxy‐coated steel. g‐i,ii) Equivalent circuit used to simulate the EIS data of coated steel. h) Tafel plots recorded in a 3.5 wt% NaCl solution for bare steel, pure epoxy‐coated steel, and 7 wt% NiAl‐LDH/G/epoxy‐coated steel. i) XRD patterns of bare steel, pure epoxy‐coated steel, and 7 wt% NiAl‐LDH/G/epoxy‐coated steel immersed in a 3.5 wt% NaCl solution for 21 days.

**Figure** **7**a–f presents the electrochemical impedance spectroscopic (EIS) data of the pure epoxy‐coated steel and 7 wt% NiAl‐LDH/G/epoxy‐coated steel. For the pure epoxy coating, the Nyquist plots show that the radius of the impedance arc decreases monotonically with the increase in the immersion time, indicating a decline in its anticorrosion performance with time (Figure 7a). For the epoxy coating with 7 wt% NiAl‐LDH/G, the radius of the impedance arc increased abruptly on day 3, exceeding that on first day (Figure 7d). This behavior can be attributed to the chloridion‐capture ability of NiAl‐LDH‐NO_3_^−^. Owing to the weak affinity of NO_3_^−^ for the LDH layers, the intercalated NO_3_^−^ ions are easily exchanged with the corrosive Cl^−^ ions in the simulated seawater; they are then trapped in the interlayers of NiAl‐LDH, improving the anticorrosion property of the coating.^[^ ^55^ ^]^ In general, the impedance modulus at 0.01 Hz (|*Z*|_0.01Hz_) is regarded as a semiquantitative indicator of the barrier performance of a coating.^[^ ^56^ ^]^ As shown in Figure 7b, the |*Z*|_0.01Hz_ value of the pure epoxy coating decreased from 5.28 × 10^3^ to 3.55 × 10^3^ Ω, indicating the decline in its barrier performance. Notably, the |*Z*|_0.01Hz_ value remained stable at ≈4.8 × 10^3^ Ω between day 5 and day 14. This is because the passivation film formed from the reaction between steel and the corrosive medium prevented the electrolyte penetration. After immersion for 21 days, the |*Z*|_0.01Hz_ value of the coating with 7 wt% NiAl‐LDH/G was still maintained at a high level of 7.82 × 10^3^ Ω, indicating its remarkable barrier performance (Figure 7e).

Usually, the frequency–phase angle diagram can be used to identify the time constant of the corrosion process. However, the time constants are difficult to distinguish from Figure 7c,f, owing to a strong overlap of the plots. In order to further investigate the electrochemical properties of the coatings, the EIS data were analyzed using an equivalent circuit (EC). The EC in Figure 7g–i was used to fit the EIS data of the pure epoxy coating. In this circuit, *R*_sol_ represents the solution resistance; *R*_por_ and CPE_por_ represent the resistance and constant phase element of the porous layer, respectively; and *R*_ct_ and CPE_dl_ are the charge‐transfer resistance and double‐layer constant phase element, respectively. For the EC of the epoxy coating with 7 wt% NiAl‐LDH/G presented in Figure 7g‐ii, the two new electrical elements, *R*_LG_ and CPE_LG_, can be assigned to the resistance and constant phase element of the NiAl‐LDH/G film. The data extracted by fitting are displayed in Tables S1 and S2 (Supporting Information), respectively. The *χ* ^2^ error values are in the order of 10^−4^–10^−3^ for all cases, implying that the chosen EC is adequate for fitting the data in Figure 7a,c.^[^ ^8^ ^]^ As an indicator of the corrosion activity at the electrolyte/substrate interface, *R*_ct_ is a vital parameter to be noted. As shown in Figure S4b (Supporting Information), the *R*_ct_ values of the coating with 7 wt% NiAl‐LDH/G are much higher than those of the pure epoxy coating during the entire duration of immersion, indicating that the introduced NiAl‐LDH/G effectively improves the anticorrosion property of the epoxy coating. In general, the *R*_ct_ value may decline with prolonged immersion. The considerable increase in the *R*_ct_ value for the 7 wt% NiAl‐LDH/G‐based coating in the initial 3 days is due to the excellent chloridion‐capture capacity of NiAl‐LDH.^[^ ^57^, ^58^ ^]^

Figure 7h shows the Tafel curves of different samples. Using the electrochemical analyzer software, the corrosion parameters, including the corrosion potential (*E*_corr_), corrosion current density (*i*_corr_), anodic Tafel slopes (*b*_a_), and cathodic Tafel slopes (*b*_c_), were calculated, and are presented in Table S3 (Supporting Information). Among the three samples, the 7 wt% NiAl‐LDH/G‐based coating shows the highest *E*_corr_ value of −381 mV and the lowest *i*_corr_ value of 1.0 × 10^−2^ mA cm^−2^. These results indicate that the 7 wt% NiAl‐LDH/G coating has a relatively weak self‐corrosive tendency, consistent with the OCP values discussed earlier.

**Figure 8:**
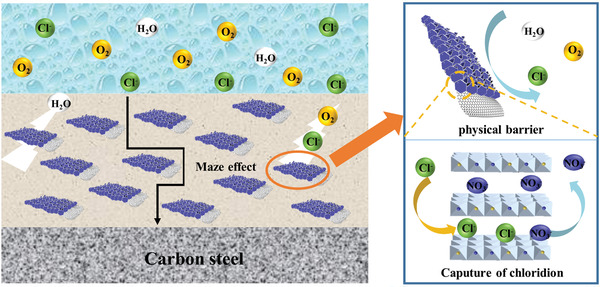
Schematic of the corrosion protection mechanism of NiAl‐LDH/G.

After a long immersion time in the 3.5 wt% NaCl solution, the corrosion products of the three samples were characterized by XRD. For bare steel, the corrosion products are mainly composed of Fe_3_O_4_ and *α*‐FeOOH. The related reactions are presented in Equations (S6)–(S10) (Supporting Information). For pure epoxy‐coated steel, the corrosive medium can permeate the coating through the pores, causing the generation of the corrosion products of Fe_2_O_3_ and *β*‐FeOOH. However, no obvious corrosion product was found in the metal/coating interface of the 7 wt% NiAl‐LDH/G/epoxy‐coated steel, suggesting its excellent anticorrosion capability. Based on all the above results, the excellent anticorrosion property of the coating with 7 wt% NiAl‐LDH/G may be attributed to the following reasons (**Figure** **8**):

1) The tiny pores of epoxy are filled by the LDH/G particles, which aid the isolation of the coating from the electrolyte. The “Maze effect” caused by the 3D structure of NiAl‐LDH/G can also afford a tortuous diffusion pathway for the corrosive medium, and thus delay the corrosion.
2) The corrosive Cl^−^ can be absorbed and trapped in the interlayers of NiAl‐LDH, owing to the anion‐exchange ability of LDH as well as the weak affinity of NO_3_^−^ for the LDH.

## 3 Conclusion

3D NiAl‐LDH/G composite was synthesized by an in situ growth strategy assisted by an ALD method, and was used as an anticorrosive microwave absorber. By controlling the content of the NiAl‐LDH nanoflakes in the composite, good impedance matching, which plays a vital role in the microwave absorption performance, was achieved. The optimized 100 NiAl‐LDH/G filler exhibited an excellent microwave absorption performance with a minimum RL value of −41.5 dB at the frequency of 17.8 GHz and a maximum EAB of 4.4 GHz at a loading of only 7 wt% in an epoxy coating. Furthermore, electrochemical measurements suggested that the coating with 7 wt% NiAl‐LDH/G can provide long‐term corrosion protection for carbon steel. The mechanism of the synergetic protection is based on the superior impermeability of graphene and the chloridion‐capture capacity of NiAl‐LDH. In addition, the 3D‐structured NiAl‐LDH/G is also beneficial for delaying the initiation of corrosion. Therefore, the NiAl‐LDH/G composite is a promising filler candidate for anticorrosive microwave absorbers. Our findings can inspire further work on the development of functional microwave absorbers that meet the demands of anticorrosive coatings.

## 4 Experimental Section

##### Preparation of Al_2_O_3_/G

The ALD process was carried out in a custom‐made, closed, hot‐wall ALD reactor. Deionized (DI) H_2_O was used as the oxidizing agent at a reactor temperature of 150 °C. Each cycle was consisted of 0.03 s of the Al(CH)_3_ pulse and another 0.2 s of the H_2_O pulse. By repeating this periodic cycle 50, 100, and 150 times, a series of samples were obtained, which were denoted as 50‐, 100‐, and 150 Al_2_O_3_/G, respectively.

##### Preparation of NiAl‐LDH/G

NiAl‐LDH‐decorated graphene was obtained by a facile hydrothermal method, as reported previously.^[^ ^59^, ^60^ ^]^ First, 0.01 mol of Ni(NO_3_)_2_·6H_2_O and 0.015 mol of NH_4_NO_3_ were dissolved in 70 mL of DI water. Then, 0.1 g of Al_2_O_3_/G was dispersed in the above solution by ultrasonic agitation (20 min). Subsequently, the mixed solution was transferred into a Teflon‐lined autoclave and heated at 100 °C for 48 h. Finally, the black products were collected by centrifugation, washed with deionized water and ethanol, and finally dried at 60 °C for 12 h. The so‐obtained products were denoted as 50‐, 100‐, and 150 NiAl‐LDH/G, respectively.

##### Characterization

The morphology was characterized by SEM (Hitachi 4800), TEM, SAED, and EDS (JEOL‐2100). The phase and crystal structure were analyzed by XRD (Bruker D8, Cu K*α* radiation), FTIR spectroscopy (TENSOR27), and XPS (AXIS SUPRA, Al K*α* source). The NiAl‐LDH to graphene weight ratio of NiAl‐LDH/G was determined by TGA (SDT Q600, 10 °C min^−1^). The BET surface area and BJH pore volumes were determined by the analyses of N_2_ absorption–desorption isotherms (TriStar II20).

The relative complex permeability and permittivity values were collected using a vector network analyzer (Agilent N5230A) by the transmission/reflection coaxial line method (2–18 GHz). The specimens used for evaluating the electromagnetic properties were prepared by uniformly mixing 7 wt% NiAl‐LDH/G samples with epoxy, and then cutting the specimen into a toroid with 7.00 mm outer diameter and 3.04 mm inner diameter. The relative computational equations for the RL, attenuation constant (*α*), impedance matching, and effective permittivity ($\varepsilon_{\mathrm{eff}}^{\mathrm{MG}}$) are presented in Equations (S1)–(S5) (Supporting Information).

The electrochemical properties were evaluated on an electrochemical workstation system (CHI660D) equipped with a typical three‐electrode system including a reference electrode (saturated calomel electrode), a counter electrode (platinum foil), and a working electrode. Bare carbon steel (45#), pure epoxy‐coated carbon steel, and 7 wt% NiAl‐LDH/G‐coated carbon steel (coating thickness, 20 µm) with test areas of 1 cm^2^ were used as the working electrodes in this work. A 3.5 wt% NaCl solution was used as the electrolyte. The polarization curves were recorded in the potential window range from −1 to 0 V at a scan rate of 10 mV s^−1^. Electrochemical impedance spectroscopy was performed in the frequency range of 100 kHz to 0.01 Hz using a sinusoidal potential perturbation of 10 mV.

## Conflict of Interest

The authors declare no conflict of interest.

## Supporting information

Supporting Information
