# Supplementary material for: Growth of NiAl‐Layered Double Hydroxide on Graphene toward Excellent Anticorrosive Microwave Absorption Application
Source: Adv Sci (Weinh). 2021 Jan 6;8(5):2002658. doi: 10.1002/advs.202002658 (PMC7927622; doi:10.1002/advs.202002658)
Supplement: Supplementary file 1 — Supporting Information [file ADVS-8-2002658-s001.pdf]

© 2020 Wiley-VCH GmbH

ADVANCED SCIENCE
Open Access

Supporting Information

for *Adv. Sci.,* DOI: 10.1002/advs.202002658

Growth of NiAl Layered Double Hydroxide on Graphene for Application as an Excellent Anticorrosive Microwave Absorber

Xuefei Xu, Shaohua Shi, Yulin Tang, Guizhen Wang, Maofan Zhou, Guoqing Zhao, Xuechun Zhou, Shiwei Lin, Fanbin Meng**

© 2020 Wiley-VCH GmbH

Supporting Information

Growth of NiAl Layered Double Hydroxide on Graphene for Application as an Excellent Anticorrosive Microwave Absorber

Xuefei Xu, Shaohua Shi, Yulin Tang, Guizhen Wang, Maofan Zhou, Guoqing Zhao, Xuechun Zhou, Shiwei Lin, Fanbin Meng**

X Xu, Dr. S Shi, Y Tang, M Zhou, G Zhao, X Zhou, Prof. G Wang, Prof. S Lin. State Key Laboratory of Advanced Materials of Tropical Island Resources (Ministry of Education), Hainan University, Haikou, Hainan, 570228, P.R. China.

Prof. F Meng, State Key Laboratory of Advanced Technologies of Materials (Ministry of Education), School of Materials Science and Engineering, Southwest Jiaotong University, Chengdu, Sichuan, 610031, P.R. China.

E-mail: wangguizhen@hainanu.edu wangguizhen0@hotmail.com (Guizhen Wang),
mengfanbin_wing@126.com (Fanbin Meng)

The reflection loss values of as-prepared samples were researched according to the transmission line theory:

$$Z_{\mathrm{in}} = Z_0\left(\frac{\mu_{\mathrm{r}}}{\varepsilon_{\mathrm{r}}}\right)\tanh\left[j\left(\frac{2\pi f d}{c}\right)(\mu_{\mathrm{r}}\varepsilon_{\mathrm{r}})^{\frac{1}{2}}\right]\#(1)$$

$$\mathrm{RL} = 20\mathrm{Log}\left|\frac{Z_{\mathrm{in}} - Z_0}{Z_{\mathrm{in}} + Z_0}\right|\#(2)$$

where Z_{in} is the input impedance of the absorber, Z_0 the impedance of free space, μ_{r} the relative complex permeability, ε_{r} the complex permittivity, f the frequency of microwaves, c the velocity of light, and d the thickness of the absorber.

The attenuation constant (α) was calculated by following equation:

$$\alpha = \frac{\sqrt{2}\pi f}{c}\times\sqrt{(\mu''\varepsilon'' - \mu'\varepsilon') + \sqrt{(\mu''\varepsilon'' - \mu'\varepsilon')^2 + (\mu'\varepsilon'' + \mu''\varepsilon')^2}}\#(3)$$

The impedance matching can be expressed as the equation:

$$Z = \frac{Z_{in}}{Z_0} = \sqrt{\left|\frac{\mu_r}{\varepsilon_r}\right|}\, tanh\left[j\left(\frac{2\pi f d}{c}\right)\sqrt{\mu_r\varepsilon_r}\right]\#(4)$$

Where Z_{in} is the impedance of microwave absorber and Z_0 is the impedance of free space. When the Z value is close to 1, microwave can enter into the absorber easily and then be converted to thermal or other energy.

The effective permittivity (ε_{eff}^{MG}) of them is highly dependent on the, which can be measured as the following equation:

$$\varepsilon_{eff}^{MG} = \varepsilon_1\frac{(\varepsilon_2 + 2\varepsilon_1) + 2f_v(\varepsilon_2 - \varepsilon_1)}{(\varepsilon_2 + 2\varepsilon_1) - f_v(\varepsilon_2 - \varepsilon_1)}\#(5)$$

Where ε_1 is permittivity of the solid, ε_2 permittivity of the void and f_v the pore volumes.

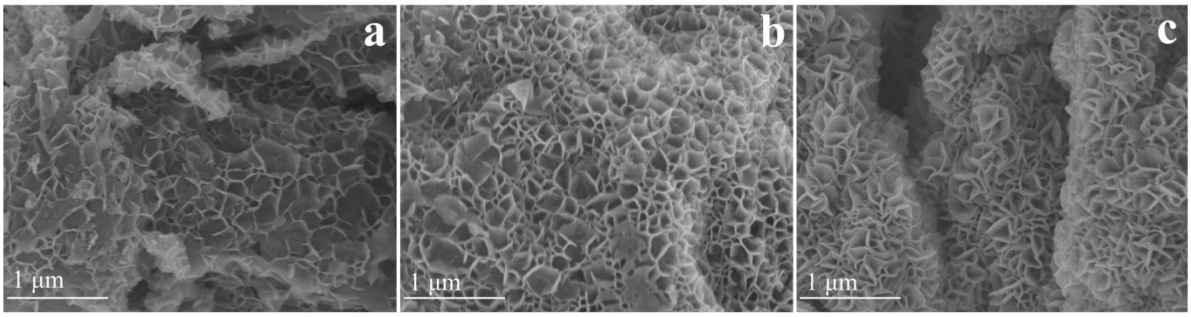

Figure S1. SEM images of (a) 50 NiAl-LDH/G, (b) 100 NiAl-LDH/G and (c) 150 NiAl-LDH/G.

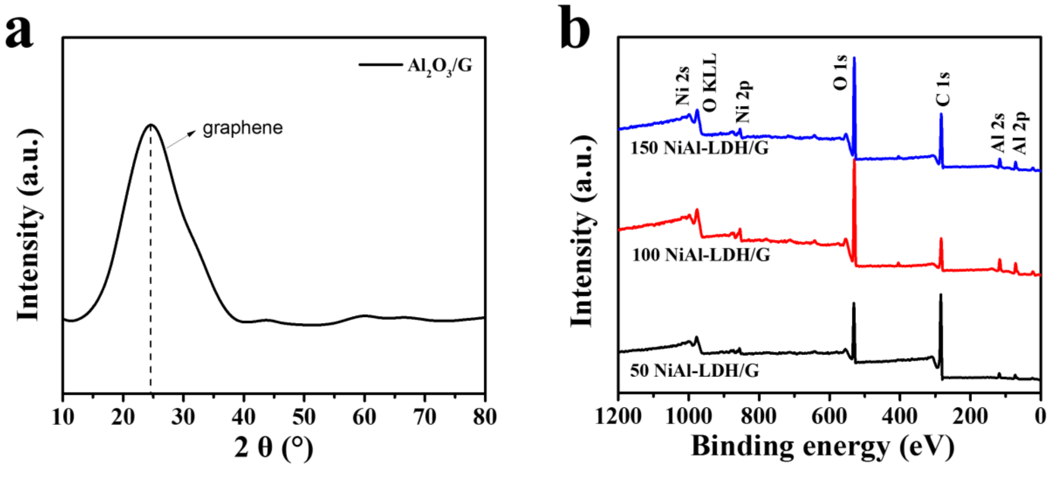

Figure S2. (a) XRD pattern of Al_2O_3/G. (b) XPS spectra of NiAl-LDH/G samples.

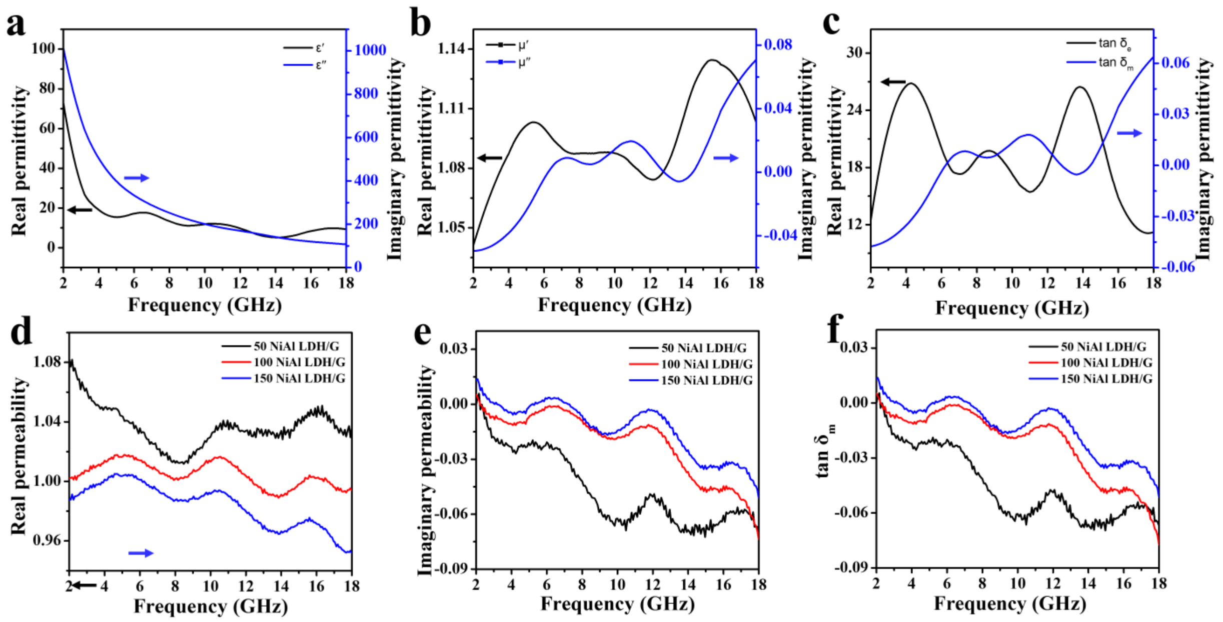

Figure S3. The frequency dependence of (a) real permittivity (ε') and imaginary permittivity (ε''), (b) real permeability (μ') and imaginary permeability (μ''), (c) dielectric loss tangent (*tan* δ_e) and Magnetic loss tangent (*tan* δ_m) of graphene. The frequency dependence of (d) real permeability (μ'), (e) imaginary permeability (μ'') and (f) magnetic loss tangent (*tan* δ_m) of NiAl-LDH/G samples.

Table S1. The electrochemical parameters fitted from EIS results of pure epoxy coated steel after immersion of different time.

Epoxy	R_{sol} (Ω cm^2)	CPE_{pro}		R_{pro} (Ω cm^2)	CPE_{dl}		R_{ct} (KΩ cm^2)	χ^2
		Y_0 (10^{-5} Ω^{-1} cm^{-2} s^n)	n		Y_0 (10^{-5} Ω^{-1} cm^{-2} s^n)	n		
1 day	49.1±0.4	12.0±1.1	0.6	25.9±3.3	3.7±0.6	0.8	5.3±0.1	1.2×10^{-3}
3 days	47.7±0.4	10.1±0.4	0.7	23.2±2.6	4.7±0.2	0.8	5.2±0.1	1.3×10^{-3}
5 days	48.6±0.4	10.5±1.2	0.7	33.4±4.0	3.8±0.7	0.8	4.5±0.1	9.3×10^{-4}
7 days	50.5±0.4	10.1±0.9	0.6	40.4±6.4	3.9±0.9	0.8	4.6±0.1	7.7×10^{-4}
14 days	48.2±0.2	11.5±0.5	0.7	50.6±7.2	3.5±0.7	0.9	4.7±0.2	4.1×10^{-4}
21 days	50.2±0.3	8.9±0.8	0.7	80.1±10.6	1.6±0.4	0.9	2.9±0.2	4.1×10^{-4}

Table S2. The electrochemical parameters fitted from EIS results of 7 wt.% NiAl-LDH/G coated steel after immersion of different time.

LDH/G	R_{sol} (Ω cm^2)	CPE_{pro}		R_{pro} (Ω cm^2)	CPE_{LG}		R_{LG} (Ω cm^2)	CPE_{dl}		R_{ct} ($K\Omega$ cm^2)	χ^2
		Y_0 (10^{-8} Ω^{-1} cm^{-2} s^n)	n		Y_0 (10^{-5} Ω^{-1} cm^{-2} s^n)	n		Yc_0 (10^{-5} Ω^{-1} cm^{-2} s^n)	n		
1 day	71.0±2.1	13.2±2.3	0.6	26.1±1.7	10.1±1.0	0.6	303.9±26.9	6.9±0.9	0.8	10.7±0.2	1.2×10^{-3}
3 days	60.3±2.2	7.4±1.1	0.6	31.5±1.7	10.7±0.8	0.6	319.9±24.6	6.7±0.6	0.8	12.4±0.2	7.5×10^{-4}
5 days	68.9±2.5	6.0±0.6	0.7	60.0±2.0	14.0±1.0	0.6	352.3±34.6	6.5±0.9	0.8	10.4±0.2	8.7×10^{-4}
7 days	54.7±1.5	11.2±1.3	0.7	28.3±1.2	14.9±1.0	0.6	296.6±33.9	6.2±0.9	0.9	10.3±0.2	7.7×10^{-4}
14 days	37.2±0.8	16.2±1.7	0.8	18.1±0.7	16.3±0.7	0.7	425.3±48.1	4.2±0.6	0.9	8.7±0.1	5.4×10^{-4}
21 days	39.5±0.9	11.3±0.9	0.6	25.2±0.8	16.3±0.7	0.7	546.7±72.0	3.2±0.6	0.9	7.9±0.1	3.6×10^{-4}

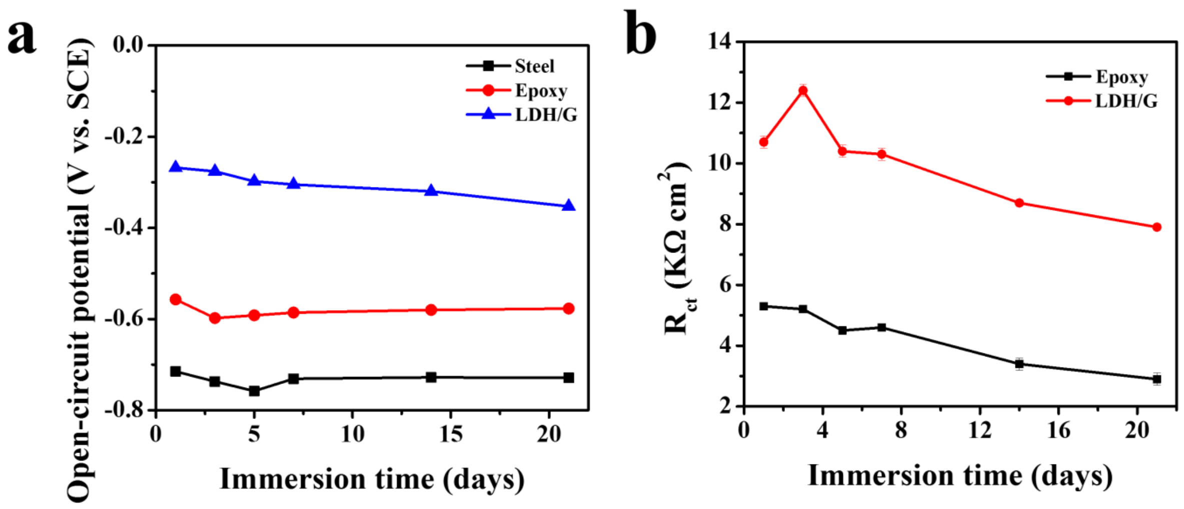

Figure S4. (a) Evolution of OCP values for bare steel, pure epoxy coated steel and 7 wt.% NiAl-ccepoxy coated steel and 7 wt.% NiAl-LDH/G coated steel with continuous immersion of 21 days.

Table S3. The parameters fitted from tafel plots results in Figure 8h.

samples	E_{corr} (mV)	i_{corr} (mA/cm^2)	b_a (mV/dec)	b_c (mV/dec)
Steel	– 926	1.2×10^{-2}	4225	– 1988
Epoxy	– 667	1.1×10^{-2}	4766	– 4852
LDH/G	– 381	1.0×10^{-2}	5854	– 5554

The corrosion process on the surface of bare carbon steel:

$$Fe \rightarrow Fe^{2+} + 2e^{-} \#(6)$$

$$Fe^{2+} \rightarrow Fe^{3+} + e^{-} \#(7)$$

$$H_2O + \frac{1}{2}O_2 + 2e^{-} \rightarrow 2OH^{-} \#(8)$$

$$2Fe^{2+} + O_2 + 2H_2O \rightarrow 2FeOOH + 2H^{+} \#(9)$$

$$Fe^{2+} + 2Fe^{3+} + 8OH^- \rightarrow Fe_3O_4 + 4H_2O \#(10)$$
